# 4-Year Follow-Up after Transatrial Transcatheter Tricuspid Valve Replacement with the LuX-Valve

**DOI:** 10.3390/jcdd9120435

**Published:** 2022-12-04

**Authors:** Xiaoping Ning, Jingyi Cao, Wei Wang, Guangwei Zhou, Fan Yang, Zhiyun Xu, Lin Han, Fan Qiao, Fanglin Lu

**Affiliations:** Department of Cardiovascular Surgery, Changhai Hospital Affiliated to the Naval Medical University, Shanghai 200433, China

**Keywords:** tricuspid valve, tricuspid regurgitation, transcatheter tricuspid valve replacement, LuX-Valve

## Abstract

Tricuspid regurgitation (TR) has become one of the most common valve diseases. Patients with severe TR are often at high surgical mortality risk. Transcatheter tricuspid valve interventions have emerged as a promising alternative to open-heart surgery. The LuX-Valve is a novel radial force-independent transcatheter tricuspid valve replacement system. We presented here the first patient treated for symptomatic TR using the LuX-Valve replacement system in September 2018. Four-year follow-up outcomes suggested that the bioprosthesis was in normal function, with stable hemodynamics (mean transtricuspid gradient 2.55 mmHg) and the patient’s clinical symptoms were significantly improved; thus indicating that it is a safe, effective, and satisfactory case of the LuX-Valve application in treating a patient with severe TR.

## 1. Introduction

Tricuspid regurgitation (TR) is a progressive disease with significant impact on mortality [1,2]. The etiology of TR is most commonly functional, due to left-sided heart disease, pulmonary hypertension, left ventricular dysfunction, or atrial fibrillation [3,4]. It is estimated that there are more than 1.6 million patients with moderate or greater TR in the United States [5]. A study on the national trends of isolated tricuspid valve (TV) surgery showed that 40.8% of patients had TV repair, and 59.2% had TV replacement surgeries performed in the United States [6]. Due to high surgical mortality for isolated TV disease [6,7], a number of transcatheter tricuspid valve intervention devices have emerged as promising alternatives to surgery [8].

**Figure 1:**
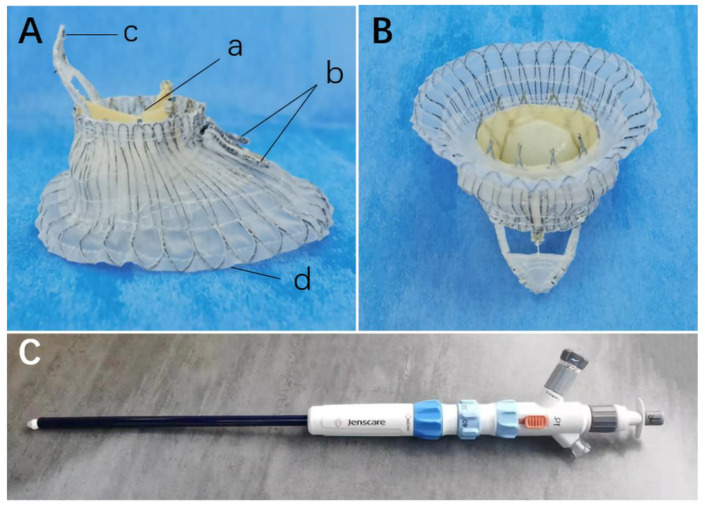
The LuX-Valve system. (**A**) The side view of LuX-Valve (a) bovine pericardial trileaflet (b) two anterior leaflet graspers (c) a bird tongue-shaped ventricle septum anchoring device (d) an atrial disc. (**B**) The atrial view of LuX-Valve. (**C**) The delivery system.

The LuX-Valve (Ningbo Jenscare Biotechnology Co., Ningbo, China) is a novel radial force-independent transcatheter tricuspid valve replacement (TTVR) system. The LuX-Valve bioprosthesis consists of a self-expandable bovine pericardial trileaflet valve, mounted in skirt-shaped nitinol valved stent, two anterior leaflet graspers, a bird tongue-shaped ventricle septum anchoring device, and an atrial disc (Figure 1A,B). The first-generation device is designed to be delivered through a transatrial approach, using a 32-F catheter (Figure 1C) [9,10]. The valve is available in a range of sizes and is suitable for patients with a maximal tricuspid annular (TA) diameter of 6.5 cm, covering almost all tricuspid regurgitation (TR) patients. Here we report the first human case with LuX-Valve and its 4-year outcomes.

## 2. Case Report

**Figure 2:**
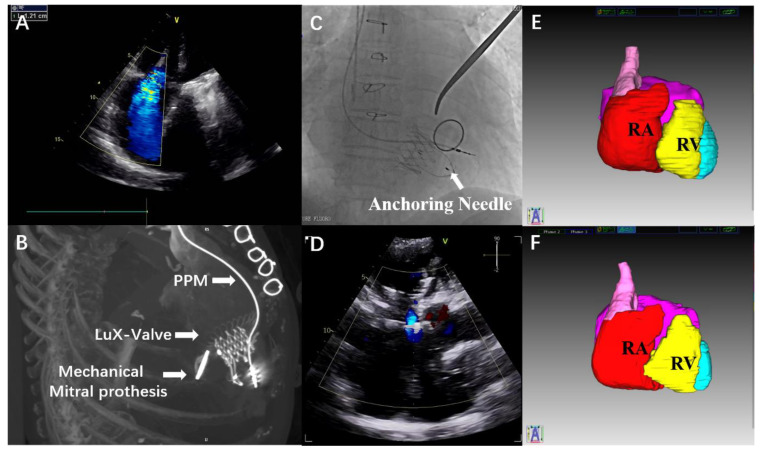
Procedural Steps of LuX-Valve Transatrial Tricuspid Valve Replacement. (**A**) Pre-operative transthoracic echocardiogram discloses severe tricuspid regurgitation. (**B**) Post-operative computed Tomography (CT) reconstruction shows the implants of the patient. (**C**) The fluoroscopy after releasing the anchoring needle. (**D**) A transthoracic echocardiogram shows trivial paravalvular leakage at 4-year follow-up. (**E**) Three-dimensional CT reconstruction of the whole heart with volumetric assessment of RV (shaded yellow). (**F**) Three-dimensional CT reconstruction indicating a slight increase in RV at 4-year follow-up. PPM = permanent pacemaker; RV = right ventricle.

The patient was a 68-year-old woman who presented with severe functional TR (vena contracta width, 12.1 mm) (Figure 2A, Video S1), severe right ventricular dysfunction, severe pulmonary hypertension (systolic pulmonary artery pressure, 61 mmHg), heart enlargement and congestive cirrhosis, with severe limitations in her daily activities. The functional capacity of this patient was in New York Heart Association (NYHA) function class IV. The patient’s history revealed a permanent pacemaker implantation 7 years ago, and a surgery of mitral valve replacement and tricuspid valve repair 12 years ago (Figure 2B). We performed a compassionate-use TTVR using the LuX-Valve system in September 2018. Pre-procedural computed tomography provided an accurate assessment of the TA, and the optimal fluoroscopic projection for the procedure.

A minimal incision was made in the right anterior lateral chest wall at the fifth intercostal space, followed by suturing two purse strings in the right atrium. Then, the delivery system was inserted into the right ventricle and centered in the TA, to ensure coaxial alignment of the valved stent before deployment, as described previously [10]. A turn knob system of the delivery system was operated to draw back the outer sheath of the delivery system gradually. The ventricle septum anchoring device was released, and then the stent valve was unfolded. Next, the two anterior leaflet graspers were positioned under the anterior leaflet, under the guidance of fluoroscopy and transesophageal echocardiography, followed by withdrawing the delivery system until the graspers successfully grasped the anterior leaflet. If the anterior leaflet was captured by the graspers, the graspers would shift with the movement of the TV. Subsequently, the atrial disc was released. The bioprosthesis could be finely adjusted to minimize paravalvular leakage, before final fixation with the anchoring needle. When we ensured that the position and the orientation were satisfactory, the anchoring needle was released through a separate channel of the delivery system and penetrated the bird tongue-shaped ventricle septum anchoring device into the ventricle septum (Figure 2C, Video S2). After immobilizing the LuX-Valve, the delivery system was drawn back, and the purse sutures were tied up.

Transesophageal echocardiography showed the correct position of the implant, with trivial paravalvular leakage. Right atrial pressure decreased from 20 mmHg preoperatively, to 14 mmHg immediately postoperatively.

At 4-year follow-up, the echocardiographic assessment indicated normal function and stable hemodynamics of the bioprosthesis (mean transtricuspid gradient 2.55 mmHg), with stable trivial paravalvular leakage (Figure 2D, Video S3). Three-dimensional computed tomography reconstruction showed a slight increase in right ventricular diastolic volume from 146.2 mL to 154.3 mL (Figure 2E,F). No device migration, three-degree atrioventricular block, or coronary artery compression occurred, largely benefiting from the advantage of the radial force-independent. During the follow-up period, the patient’s clinical symptoms were significantly improved, with NYHA function class in II.

## 3. Discussion

TR was overlooked in the past, and researchers have focused on the treatment of this disease in recent decades. Surgical treatment is often faced with high in-hospital mortality and postoperative complications [7], so the academic community, naturally, began to explore interventional treatment. Transcatheter tricuspid valve interventions (TTVIs) can be categorized into transcatheter tricuspid valve repair (TTVr), which includes coaptation devices and annuloplasty devices, TTVR, and caval valve implantation (CAVI). With TTVr it is often difficult to completely eliminate the regurgitation, and it is easy to relapse. Additionally, it has higher requirements of anatomy and pathology. TTVR can effectively eliminate the regurgitation; however, heterotopic CAVI can only prevent cava from backflow. The tricuspid valve itself has no obvious change, and the volume overload of the right ventricle persists. TTVR devices seem to be more suitable for the tricuspid valve anatomy than other devices.

There are few existing TTVR devices, mainly including NaviGate, EVOQUE System, LuX-Valve, Cardiovalve, and CroíValve. All these TTVR devices are radial force-dependent devices except for the LuX-Valve. Clinical trials show that TTVR devices can effectively eliminate TR and reduce right atrial pressure [11,12]. However, due to the limited valve size, and the occurrence of complications of these radial force-dependent devices, it is difficult for some devices to obtain satisfying clinical outcomes when treating patients with oversized TA. The radial force-independent and skirt-shaped design of the LuX-Valve allows it to accommodate different sizes of annulus, and reduces the occurrence of complications such as third-degree atrioventricular block.

## 4. Conclusions

This report shows a successful case of LuX-Valve on a medium to long-term follow-up patient. It is a safe, effective, and satisfactory case of the LuX-Valve application in treating a patient with severe TR. A multicenter, long-term study is ongoing to further evaluate the safety of the device (TRAVEL study, NCT04436653).

## Data Availability

Data were uploaded as suggested by Data Availability Statements in section “MDPI Research Data Policies”.

## Supplementary Materials

The following supporting information can be downloaded at: https://www.mdpi.com/article/10.3390/jcdd9120435/s1.
